# Association of HbA1c Values with Mortality and Cardiovascular Events in Diabetic Dialysis Patients. The INVOR Study and Review of the Literature

**DOI:** 10.1371/journal.pone.0020093

**Published:** 2011-05-18

**Authors:** Gisela Sturm, Claudia Lamina, Emanuel Zitt, Karl Lhotta, Florian Haider, Ulrich Neyer, Florian Kronenberg

**Affiliations:** 1 Division of Genetic Epidemiology, Department of Medical Genetics, Molecular and Clinical Pharmacology, Innsbruck Medical University, Innsbruck, Austria; 2 Department of Nephrology and Dialysis, Academic Teaching Hospital Feldkirch, Feldkirch, Austria; 3 Vorarlberg Institute for Vascular Investigation and Treatment (VIVIT), Feldkirch, Austria; German Diabetes Center, Leibniz Center for Diabetes Research at Heinrich Heine University, Germany

## Abstract

**Background:**

Improved glycemic control reduces complications in patients with diabetes mellitus (DM). However, it is discussed controversially whether patients with diabetes mellitus and end-stage renal disease benefit from strict glycemic control.

**Methods:**

We followed 78 patients with DM initiating dialysis treatment of the region of Vorarlberg in a prospective cohort study applying a time-dependent Cox regression analysis using all measured laboratory values for up to more than seven years. This resulted in 880 HbA_1c_ measurements (with one measurement every 3.16 patient months on average) during the entire observation period. Non-linear P-splines were used to allow flexible modeling of the association with mortality and cardiovascular disease (CVD) events.

**Results:**

We observed a decreased mortality risk with increasing HbA_1c_ values (HR = 0.72 per 1% increase, p = 0.024). Adjustment for age and sex and additional adjustment for other CVD risk factors only slightly attenuated the association (HR = 0.71, p = 0.044). A non-linear P-spline showed that the association did not follow a fully linear pattern with a highly significant non-linear component (p = 0.001) with an increased risk of all-cause mortality for HbA_1c_ values up to 6–7%. Causes of death were associated with HbA_1c_ values. The risk for CVD events, however, increased with increasing HbA_1c_ values (HR = 1.24 per 1% increase, p = 0.048) but vanished after extended adjustments.

**Conclusions:**

This study considered the entire information collected on HbA_1c_ over a period of more than seven years. Besides the methodological advantages our data indicate a significant inverse association between HbA_1c_ levels and all-cause mortality. However, for CVD events no significant association could be found.

## Introduction

It is well documented that improved glycemic control reduces complications in patients with diabetes mellitus (DM) [1], however, it is not clear whether patients with DM and end-stage renal disease (ESRD) benefit from strict glycemic control [2]. NKF-K/DOQI guidelines recommend a target HbA_1c_ of <7% for patients with DM and chronic kidney disease [3].

A prospective interventional study in patients with DM but without renal failure showed an increase in all-cause mortality in patients with HbA_1c_ <6% attained by intensive therapy compared to the standard therapy group [4]. Nonetheless some small observational studies mostly performed in Asian populations indicate the importance of good glycemic control for survival in dialysis patients with DM [5]–[9]. One observational study from Germany found higher HbA_1c_ values to be a risk factor for all-cause mortality and cardiovascular disease [10]. However, in several studies no association between HbA_1c_ and neither patient survival [11]–[15] nor cardiovascular disease [12] could be shown in dialysis patients with DM. Most of these studies were based on a single measurement of HbA_1c_ values. Only two studies considered time-dependent analyses using all available measurements of HbA_1c_ during the whole observation period instead of using only a baseline measurement [13], [16].

Our single-center study aimed to investigate the association of HbA_1c_ values with mortality in a prospective observational inception cohort of 78 dialysis patients with DM initiating dialysis treatment and followed for a period of up to more than seven years. To consider the broad spectrum of intraindividual variability of metabolic disturbances over time, HbA_1c_ levels as well as all other covariates recorded during the entire observation period were considered in the time-dependent Cox regression modeling. This resulted in 880 HbA_1c_ measurements during the entire observation period which were used in the analysis. Furthermore, non-linear P-splines were applied to allow flexible modeling of the association with mortality, CVD events and the combination of CVD and peripheral arterial disease (PAD) events. Our study is up to now the only inception cohort study with time-dependent measurements over a long observation period.

## Methods

### INVOR-Study

The INVOR-Study [17] (Study of Incident Dialysis Patients in Vorarlberg) is a single-center, prospective, observational cohort study of incident Caucasian hemodialysis and peritoneal dialysis patients in Vorarlberg, the westernmost province of Austria counting approximately 400,000 inhabitants.

Ethic statement: The study was approved by the ethics committee of the Innsbruck Medical University and all patients enrolled in the study provided written informed consent.

All dialysis patients from this province starting chronic dialysis treatment between May 1st, 2000 and April 30th, 2006 were consecutively enrolled with the advantage that all patients of this region are treated by the same care provider. During this period of 6 years a total number of 235 incident dialysis patients were included and followed until the study endpoint was reached or follow-up was censored at December 31st, 2007. Ten patients having a malignant tumor at initiation of dialysis were not recruited defined by the exclusion criteria. 82 out of 235 patients were diagnosed with DM at baseline, 4 of them died shortly after initiation of dialysis without having a sufficient number of HbA_1c_ measurements. Therefore 78 patients were considered for analyses, 73 of them diagnosed with DM type 2 and 5 of them with DM type 1. All data and analyses described in this manuscript are based on these 78 patients.

Patients were treated according to the European Best Practice Guidelines in place at the time of treatment (http://www.ndt-educational.org/guidelines.asp).

### Data description

As described recently clinical, laboratory and medication data were collected prospectively starting at the time of initiation of dialysis [17], [18]. Laboratory parameters were recorded continuously during the study period and measured in a central laboratory. They were measured at different time intervals, most of them once to twice monthly (hemoglobin, erythrocytes, creatinine, calcium and phosphorus), or every 3 months (HbA_1c_, albumin, C-reactive protein and ferritin). The patients had a median number of 10 HbA_1c_ measurements in the follow-up period (with a minimum of 1 and a maximum of 43 mesureaments) resulting in 880 different HbA_1c_ measurements during the entire observation period and one measurement every 3.16 patient months on average. Quality control is conducted twice a year where the laboratory takes part at the trial of the Austrian Society for Quality assurance and standardization of diagnostic medical tests (ÖQUASTA). The methodology was evaluated in our center regarding CLSI (Clinical and Laboratory Standards Institute) Evaluation Protocol 10. The intra-assay variability was 1.04% for HbA_1c_ of 5.3, 0.79% for 8.3 and 1.46% for 12.5. All available measurements of HbA_1c_ and the other variables were used in the time-dependent Cox regression modeling described below.

### Study Outcomes

The outcomes of interest were all-cause mortality as well as CVD events and the combination of CVD and PAD events. CVD events were defined as fatal and non-fatal myocardial infarction (diagnosed by clinical appearance, electrocardiography, increase in troponin T and CK-MB and in most cases followed by angiograpgy or autopsy in fatal cases), percutaneous transluminal coronary angioplasty, aortocoronary bypass, angiographically proven coronary stenosis ≥50%, sudden cardiac death, ischemic or hemorrhagic cerebral infarction (diagnosed by clinical appearance, CT or MRI or autopsy), transient ischemic attack, carotid stenosis and carotid endarterectomy). Death causes were autopsy-proven in 27% of the present patient sample. For PAD at least one of the following events was existent: significant ultrasound- or angiographically-proven vascular stenosis, percutaneous transluminal angioplasty, peripheral bypass or amputation. An incident PAD event was only considered as a first time manifestation or a deterioration of PAD in terms of e.g. a change in PAD stage according to Fontaine. One patient was lost to follow-up because of regaining renal function.

### Statistical Methods

At baseline, categorical data were compared using χ^2^-test, continuous variables were analyzed using an unpaired T-test or the nonparametric Mann-Whitney-U-test. Associations between all measured HbA_1c_ values <7% and various parameters we investigated using linear mixed effects models. To investigate the influence of HbA_1c_ levels on all-cause mortality, a time-dependent Cox Proportional Hazards model was used allowing all variables to vary over different measurements during the whole observation time for each patient. That is, each time-span between two successive measurements enters the model independently. Each covariate that entered the model was updated at the time they were measured and modeled in a time-dependent fashion. If not all variables were measured at a particular date, the respective missing values were replaced by the values measured at the last observation of this variable (“last observation carried forward”). To account for possible correlation of values within one patient robust variances were estimated, which were grouped for each patient. The Proportional Hazards assumption was tested for each model by testing for zero slopes of scaled Schoenfeld residuals.

At first, HbA_1c_ was included linearly in the model with hazard ratios referring to 1% increase. In order to evaluate the functional form of the HbA_1c_ effects, non-linear P-splines of degree 3 were estimated. A spline of degree 3 is a linear combination of cubic functions, which can fit virtually any smooth curve to the data. Therefore, the analysis was not restricted to a potential linear relationship of HbA_1c_ with risk of mortality. To keep the number of parameters estimated at a minimum, the minimum number of knots for a non-linear P-spline was chosen (df = 2). The spline term can be split into its linear and non-linear components, which can each be tested separately. For the linear term, a Hazard Ratio (HR) can be estimated, whereas the non-linear component can be depicted in a plot of the log(HR).

Cox models were calculated univariately including the time-dependent HbA_1c_ measurements and additionally adjusted for age and sex. An extended model was also conducted, additionally adjusting for CVD events before start of renal replacement therapy and the time-dependent variables systolic and diastolic blood pressure, hemoglobin, C-reactive protein, albumin. Due to the high correlation of these factors with inflammation and malnutrition, adding them as proxies in the model can adjust the presence of inflammation and malnutrition partly. We also performed a sensitivity analysis with censoring at the time of transplantation. All analyses were conducted in R using the package “survival”.

## Results

Table 1 presents the baseline demographic and laboratory characteristics as well as comorbidities before the start of dialysis treatment of 78 patients with DM (46 men and 32 women). The median follow-up time was 31.9 months. During this period, 33 patients died (42.3%). 17 patients died of cardiovascular disease (51.0% of the death causes), 8 died of a fatal sepsis (25.5%) and 8 patients had other causes of mortality (23.5%).

**Table 1:** Clinical characteristics of patients at baseline and during follow-up stratified by survival and by CVD events.

	All patients	Survivors	Non-Survivors	No CVD events	CVD events**
	(n = 78)	(n = 45)	(n = 33)	(n = 40)	(n = 38)
Sex (male/female), n (%)	46/32 (59/41%)	28/17 (62/38%)	18/15 (55/45%)	23/17 (58/43%)	23/15 (61/39%)
Age (years)	65.5±9.2	65.3±9.2	65.8±9.3	68.8±9.3	62.1±7.8^c^
Body Mass Index (kg/m^2^)	27.8±4.5	28.2±4.0	27.2±5.1	27.4±4.8	28.2±4.2
Start of dialysis with					
Hemodialysis, n (%)	73 (94%)	45 (100%)	28 (85%)^a^	38 (95%)	35 (92%)
Central venous catheter, n (%)	12 (16%)	6 (13%)	6 (21%)	9 (24%)	3 (9%)
Native fistula, n (%)	49 (67%)	29 (64%)	20 (71%)	23 (61%)	26 (74%)
Graft, n (%)	12 (16%)	10 (22%)	2 (7%)	6 (16%)	6 (17%)
Peritoneal dialysis, n (%)	5 (6%)	0 (0%)	5 (15%)^a^	2 (5%)	3 (8%)
Systolic blood pressure (mmHg)	159±24	160±23	158±26	158±22	160±27
Diastolic blood pressure (mmHg)	82±13	84±13	79±13	80±12	84±14
Duration of diabetes mellitus (years)	16.0±10.7	15.0±9.7	17.5±11.9	14.5±9.2	17.7±12.0
***Laboratory parameters***					
HbA_1c_ (% Hb) at initiation of dialysis	7.11±1.55	6.94±1.42	7.32±1.70	6.73±1.10	7.54±1.86^a^
3 months after initiation of dialysis	7.32±1.42	7.31±1.36	7.33±1.52	6.80±0.98	7.79±1.59^c^
Albumin (g/dL)	3.6±0.6	3.7±0.5	3.5±0.5	3.4±0.6	3.7±0.4^a^
C-reactive protein (mg/dL)	3.0±4.5 [0.4; 1.1; 3.1]	3.3±4.6 [0.4; 1.2; 4.7]	2.6±4.4 [0.3; 0.9; 2.2]	3.6±5.1 [0.6; 1.6; 4.4]	2.4±3.9 [0.3; 0.8; 2.2]
Phosphorus (mmol/L)	2.01±0.63	1.97±0.66	2.07±0.60	2.01±0.73	2.01±0.52
Hemoglobin (g/dL)	11.2±1.6	11.4±1.6	11.0±1.6	11.1±1.5	11.3±1.6
Creatinine (mg/dL)	6.4±2.4	6.2±1.9	6.6±2.9	6.0±1.8	6.7±2.8
Ferritin (ng/mL)	157±165 [51; 116; 196]	154±159 [53; 112; 194]	161±175 [44; 137; 200]	178±189 [40; 149; 207]	134±132 [61; 111; 171]
***Comorbidities before dialysis***					
CAD events*, n (%)	17 (22%)	9 (20%)	8 (24%)	9 (23%)	8 (21%)
CVD events**, n (%)	33 (42%)	17 (38%)	16 (49%)	14 (35%)	19 (50%)
PAD events***, n (%)	22 (28%)	13 (29%)	9 (27%)	13 (33%)	9 (24%)
***Follow-up***					
Follow-up time (months)‡	35.6±22.1	43.3±21.6	25.1±18.4^d^	31.1±20.3	40.4±23.2
Transplantation, n (%)	7 (9%)	7 (16%)	0 (0%)^a^	5 (13%)	2 (5%)

Mean ± SD [25., 50. und 75. percentile in case of non-normal distribution] or number (%).

^***a***^p<0.05;

^***b***^p<0.01;

^***c***^p<0.005;

^***d***^p<0.001 – comparison between survivors and non-survivors as well as between patients with and without cardiovascular disease events.

*Coronary artery disease events: myocardial infarction, percutaneous transluminal coronary angioplasty, aortocoronary bypass.

**Cardiovascular disease events: myocardial infarction, percutaneous transluminal coronary angioplasty, aortocoronary bypass, angiographically-proven coronary stenosis ≥50%, ischemic or hemorrhagic cerebral infarction, transient ischemic attack, carotid stenosis and carotid endarterectomy.

***Peripheral arterial disease events: significant ultrasound- or angiographically-proven vascular stenosis, percutaneous transluminal angioplasty, peripheral bypass, amputation.

‡Follow-up time was calculated as the time from the start of dialysis until the patient died or the end of the observation period was reached.

**Figure 1:**
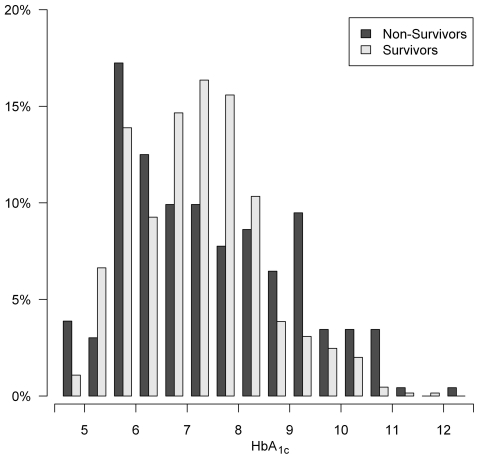
Distribution of all 880 measured HbA_1c_ values during follow-up stratified for survivors and non-survivors.

Baseline laboratory parameters did not differ significantly between survivors and non-survivors. The median duration of diabetes mellitus was 15 years. HbA_1c_ values were slightly lower in survivors than in non-survivors, if only the measurements at start of dialysis were considered, however, this difference was not significant. If measurements 3 months after start of dialysis were considered, HbA_1c_ values were very similar in survivors and non-survivors. Taking all measurements during the whole observation period into account, HbA_1c_ values were also lower in survivors compared to non-survivors (mean [95% CI]: 7.01 [6.98; 7.05] vs. 7.36 [7.30; 7.42]. Figure 1 shows the distribution of all measured HbA_1c_ values in the two groups.

To explore the death causes in relation to the HbA_1c_ values at baseline and at the early observation period, we stratified patients according to their baseline HbA_1c_ values with a threshold of 7.0% (Table 2). The 15 patients who died during the follow-up period and having HbA_1c_ values below 7% at baseline died mainly due to therapy withdrawal (33%) and heart failure (27%). Two patients stopped treatment because of sepsis, one patient due to end-stage cancer, one patient due to an ischemic stroke and one patient suffered from dementia. Only minor changes in HbA_1c_ values were observed between the baseline levels and the next measured HbA_1c_ level in these patients. In contrast, patients who died and had HbA_1c_ levels equal or above 7.0% at baseline died mainly due to sepsis (33%), myocardial infarction (22%) or sudden cardiac death (22%) (Table 2).

**Table 2:** Causes of death stratified by HbA_1c_ <7% vs. ≥7% at baseline.

	HbA_1c_ at baseline (n = 33)	
Causes of death	HbA_1c_ <7% (n = 15)	HbA_1c_ ≥7% (n = 18)
Myocardial infarction	2 (13%)	4 (22%)
Heart failure	4 (27%)	1 (6%)
Sudden cardiac death	1 (7%)	4 (22%)
Stroke	0 (0%)	1 (6%)
Sepsis/infection	2 (13%)	6 (33%)
Therapy withdrawal	5 (33%)	1 (6%)
End stage cancer	1 (7%)	0 (0%)
Other/unknown	0 (0%)	1 (6%)

We did not observe a strong linear association between HbA_1c_ values and parameters of malnutrition such as albumin (r = 0.033, p = 0.14), phosphorus (r = 0.04, p = 0.025) or CRP (r = −0.002, p = 0.40). However, when we considered the association of HbA_1c_ below 7% with these parameters, we observed that lower albumin and phosphorus concentrations and higher CRP values were associated with HbA_1c_ values below 7% (Table 3).

**Table 3:** Results from a linear mixed effects model of HbA_1c_ <7% on parameters of malnutrition and inflammation.

	Effect estimate β	P-value
***Association of HbA_1c_ <7% with***		
Albumin (g/dL)	−0.068	<0.001
C-reactive protein (mg/dL)	0.326	0.008
Phosphorus (mmol/L)	−0.062	0.001

Patients who experienced a cardiovascular disease event during the observation period had significantly higher HbA_1c_ and albumin values at the baseline investigation before dialysis treatment was started. HbA_1c_ measurements were even higher when all measurements during the whole observation period were considered (mean [95% CI]): 7.67 [7.15; 8.18] vs. 7.07 [7.04; 7.12].

### Cox regression analysis

**Figure 2:**
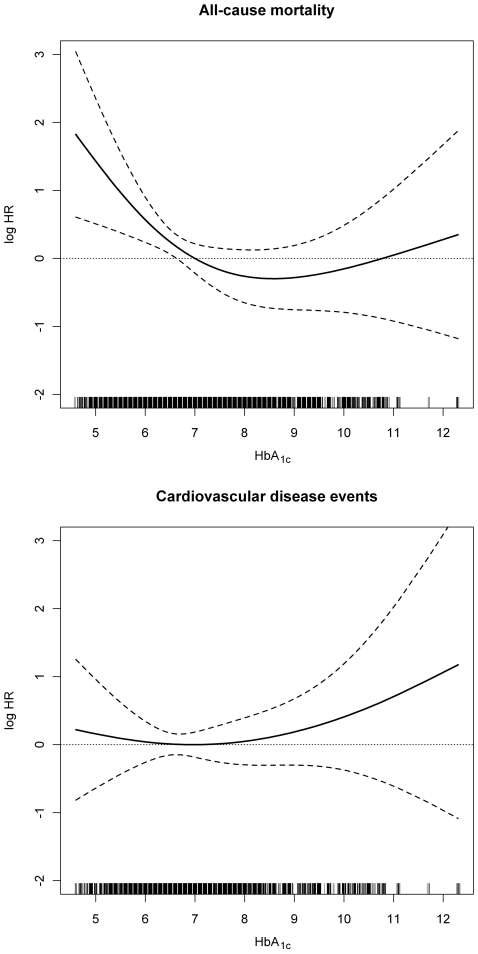
Cox regression results: P-splines to explore the functional form of the effect of HbA_1c_ values on the log hazard ratio for the risk of a) all-cause mortality and of b) cardiovascular disease events, adjusted for age, sex, time-dependent systolic blood pressure, diastolic blood pressure, albumin, CRP, hemoglobin and previous CVD. Dashed lines are the pointwise 95% CI. The rugplot at the bottom of the figures displays the number of measurements.

To make use of all information available from the entire observation period, we considered HbA_1c_ values as well as other laboratory measurements from the entire observation period in time-dependent regression models. This has the advantage that the association is not based on a single baseline measurement but on the glycemic control over the entire time of observation. Models were calculated unadjusted, age- and sex-adjusted and with an extended adjustment for blood pressure, albumin, CRP, hemoglobin and previous CVD events. We observed a significant inverse association between time-dependent HbA_1c_ measurements and all-cause mortality: the mortality risk decreased with increasing HbA_1c_ values (HR = 0.72 per 1% increase, p = 0.024). After adjustment for age and sex and additional adjustment the association was slightly attenuated but still significant (HR = 0.71 per 1% increase, p = 0.044) (Table 4). A non-linear P-spline on the fully adjusted model showed this significant relationship between decreasing HbA_1c_ values and increasing risk of all-cause mortality (Figure 2). The linear and the non-linear component of the non-linear P-spline (p = 0.034 and p = 0.001) were significant (Table 4), which implies that there is an overall negative trend, but that a simple linear model resulting in one HR would not be sufficient to describe the relationship between HbA_1c_ values and time to event. Figure 2 showed a decreasing trend up to HbA_1c_ values of ∼7%, which flattens afterwards. This deviation from linearity is depicted by the test of the non-linear part.

**Table 4:** The association of time-dependent HbA_1c_ with all-cause mortality, CVD events and the combination of CVD and PAD events using multiple Cox-proportional hazards models.

			All-cause mortality			CVD events**			CVD and PAD events***		
			(n events = 33)			(n events = 38)			(n events = 52)		
			HR	(95%CI)	P-value	HR	(95%CI)	P-value	HR	(95%CI)	P-value
**Linear effect modeling**											
Adjustment:	None		0.72	(0.54, 0.96)	0.024	1.24	(1.00, 1.54)	0.048	1.13	(0.95, 1.34)	0.164
	Age, sex		0.71	(0.53, 0.95)	0.020	1.14	(0.87, 1.50)	0.338	1.05	(0.85, 1.29)	0.664
	Extended*		0.71	(0.51, 0.99)	0.044	1.09	(0.82, 1.46)	0.554	1.04	(0.84, 1.28)	0.735
**Non-linear effect modeling using P-splines**											
Adjustment:	None	Linear part	0.70	(0.58, 0.85)	<0.001	1.20	(0.99, 1.46)	0.063	1.10	(0.93, 1.26)	0.280
		Non-linear part			0.007			0.039			0.070
	Age, sex	Linear part	0.69	(0.56, 0.84)	<0.001	1.10	(0.87, 1.38)	0.420	1.01	(0.82, 1.21)	0.890
		Non-linear part			0.008			0.074			0.094
	Extended*	Linear part	0.81	(0.66, 0.98)	0.034	1.05	(0.83, 1.33)	0.680	1.01	(0.82, 1.20)	0.900
		Non-linear part			0.001			0.110			0.170

For each model, estimated HRs are shown for the linear component of the non-linear P-spline as well as HRs for HbA_1c_ measurements per 1% increase.

*Adjusted for age, sex, time-dependent systolic blood pressure, diastolic blood pressure, albumin, CRP, hemoglobin and previous CVD **.

**Cardiovascular disease events: myocardial infarction, percutaneous transluminal coronary angioplasty, aortocoronary bypass, angiographically-proven coronary stenosis ≥50%, ischemic or hemorrhagic cerebral infarction, transient ischemic attack, carotid stenosis and carotid endarterectomy.

***Cardiovascular and peripheral arterial disease events: CVD events or significant ultrasound- or angiographically-proven vascular stenosis, percutaneous transluminal angioplasty, peripheral bypass, amputation.

Furthermore, a borderline significant association could be found between HbA_1c_ measurements and CVD events. In contrast to all-cause mortality, the risk for CVD events increased with increasing HbA_1c_ values (HR = 1.24 per 1% increase, p = 0.048). The association vanished after adjusting for age and sex as well as additional parameters (Table 4). A non-linear P-spline on the fully adjusted model showed a trend for higher HbA_1c_ values towards an increasing risk of CVD events, but neither the linear nor the non-linear component of the P-spline were significant (Figure 2 and Table 4). There was no significant association between HbA_1c_ values and the combination of CVD and PAD events (Table 4).

### Sensitivity analysis

We performed a sensitivity analysis with censoring at the time of transplantation which did, however, not reveal any substantial differences in HRs compared to the primary analysis. The unadjusted HR for all-cause mortality was 0.74 per 1% increase (p = 0.035), after adjustment for age and sex 0.73 (p = 0.027) and the HR was still significant after full adjustment (HR = 0.72 per 1% increase, p = 0.044). The non-linear component of the non-linear P-spline was significant (p = 0.002), the linear component was borderline significant (p = 0.052). We calculated a model with additionally adjusting for BMI at baseline, which did not reveal any major changes in HRs compared to the original extended model. Two further sensitivity analyses were calculated where the extended model was additionally adjusted for current smoking and phosphorus levels, respectively. No substantial differences in HRs for HbA_1c_ could be observed.

## Discussion

The study at hand used a time-dependent Cox regression analysis of a single-center inception cohort of dialysis patients with DM initiating dialysis treatment followed for up to more than seven years. It used all information of HbA_1c_ levels available from the entire observation period to model the association of HbA_1c_ levels and all-cause mortality as well as CVD events and the combination of CVD and PAD events. We observed an increased risk of mortality for lower HbA_1c_ values, but no association was found for higher HbA_1c_ levels. Furthermore, the risk for CVD events increased with higher HbA_1c_ values, but lacking statistical significance.

The ACCORD trial [4], a prospective interventional study in 10,251 patients with DM without renal failure, investigated whether HbA_1c_ of <6%, to be attained by intensive glucose control, reduces CVD events and mortality. Surprisingly, they found an increase in all-cause mortality in the intensive therapy group compared to the standard therapy group. However, the reduction in the primary outcome of nonfatal myocardial infarction, nonfatal stroke or death from cardiovascular cause with intensive therapy was not significant. It is unclear whether dialysis patients with DM benefit from strict glycemic control [2]. Observational studies reported conflicting results concerning glycemic control and large clinical trials have not been performed in this patient population [19]. All studies investigating the association between HbA_1c_ and different clinical outcomes in dialysis patients with DM are listed in Table 5 (studies which found an association) and Table 6 (studies which did not find an association). These studies were heterogeneous in several ways. Firstly, some of them investigated a linear association of HbA_1c_ values with outcomes assuming a linear “dosage” association between diabetic control and outcomes [5], [10], [11]. Other studies compared the risk for outcomes above and below a certain threshold which was either the median or mean of the investigated patient population [5], [6], [8], [12] or a group-wise comparison of the subjects above a certain threshold (poor controlled patients) against a “well controlled” group [7], [9], [10], [13]–[16], [20]. These thresholds varied widely and did sometimes neglect the main group of averaged controlled patients. To avoid this linear “dosage” assumption we used in our analysis non-linear P-splines which allow modeling of the association without a priori assumptions of thresholds. Our results point out that especially the low HbA_1c_ values are associated with an increased risk for mortality.

**Table 5:** Studies in dialysis patients with diabetes mellitus which found an association between HbA_1c_ and different clinical outcomes.

Study	Design	Follow-up	Endpoint (number of patients with endpoint): HR (95% CI)
**Drechsler et al.**	Observational cohort study:	4 yrs.	**a) HbA_1c_ >8% vs. HbA_1c_ ≤6%; b) per 1% HbA_1c_ increase; multivariable adjustment.**
2010 [10]	1255 German HD patients		CVD (n = 469): a) HR = 1.37 (1.00–1.87); b) HR = 1.09 (1.01–1.18)
			All-cause mortality (n = 617): a) HR = 1.34 (1.02–1.76); b) HR = 1.09 (1.02–1.17)
			Sudden death (n = 160): a) HR = 2.26 (1.33–3.85); b) HR = 1.21 (1.06–1.38)
			MI (n = 200): a) HR = 0.77 (0.47–1.26)
			Stroke (n = 103): a) HR = 1.67 (0.84–3.30)
			Heart failure death (n = 41): a) HR = 2.12 (0.75–5.98)
**Ishimura et al.** 2009 [5]	Observational cohort study:	55.5 mos.	**a) HbA_1c_ ≥6.3% vs. HbA_1c_ <6.3%; b) per 1% increase; adjusted for age, sex, duration of HD.**
	122 Japanese HD patients		All-cause mortality (n = 37): a) HR = 2.879 (1.439–5.759)
			CV mortality n = 19): a) HR = 2.749 (1.064–7.089)
			Non-CV mortality (n = 18): a) HR = 3.196 (1.171–8.724); b) HR = 1.418 (1.063–1.892)
**Kalantar-Zadeh et al.**	Prospective cohort study:	3 yrs.	All-cause mortality*, unadjusted: HR = 0.87 (0.82–0.89) for HbA_1c_ >6% vs.≤6%
2007 [16]	23,618 US HD patients		All-cause mortality*, multivariate adjustment: HR = 1.05 (1.01–1.10) for HbA_1c_ >6% vs. ≤6%
			CV mortality*, multivariate adjustment: HR = 1.73 (1.44–2.08) for HbA_1c_ ≥10% vs. 5.0–5.9%)
**Morioka et al.** 2001 [6]	Prospective cohort study: 150 Japanese incident HD patients	2.7 yrs.	All-cause mortality (n = 72): HR = 1.13 (1.028–1.249) for HbA_1c_ ≥7.5% vs.HbA_1c_ <7.5%; adjusted for age and sex.
**Oomichi et al.** 2006 [7]	Observational cohort study: 114 Japanese HD patients	45.5 mos.	All-cause mortality (n = 72): HR = 2.89 (1.538–5.429) for HbA_1c_ ≥8% vs. HbA_1c_ <6.5%; adjusted for age, sex, duration of HD, CVD.
**Tsujimoto et al.** 2009 [8]	Prospective cohort study: 134 Japanese HD patients	5 yrs.	CVD (n = 50): HR = 1.828 (1.008–3.314) for HbA_1c_ ≥7% compared with HbA_1c_ <7%; adjusted for age, sex, duration of HD, CVD.
**Williams et al.** 2009 [20]	Retrospective cohort study: 23,829 US HD patients	1 yr.	Hospitalization risk (71.2%): Association only with extremes of HbA_1c_ (<5 and >11%); multivariable adjustment.
**Wu et al.** 1997 [9]	Retrospective cohort study: 137 Taiwanese HD patients	1–5 yrs.	All-cause mortality (n = 48): HR = 0.37 (0.175–0.795) for HbA_1c_ <10% compared with HbA_1c_ ≥10%; adjusted for age, albumin and cholesterol.

CV, cardiovascular; CVD cardiovascular disease; MI myocardial infarction; HD hemodialysis.

*exact numbers of events are not available.

**Table 6:** Studies in dialysis patients with diabetes mellitus which did not find an association between HbA_1c_ and different clinical outcomes.

Study	Design	Follow-up	Endpoint (number of patients with endpoint): HR (95% CI)
**Fukuoka et al.** 2008 [11]	Prospective cohort study:	47.7 mos.	**per 1% HbA_1c_ increase; adjusted for age, sex, total cholesterol, CRP and albumin.**
	98 Japanese HD patients		All-cause mortality (n = 51): HR = 0.929 (0.734–1.175)
			CV mortality (n = 22): HR = 1.345 (0.867–2.086)
			Infectious death (n = 16): HR = 1.078 (0.696–1.689)
**McMurray et al.** 2002 [21]	Non-randomized trial: 83 US HD patients	1 yr.	Quality of life: no survival benefit; HbA_1c_ levels significantly decreased and quality of life was significantly improved in the study group;
**Okada et al.** 2007 [12]	Prospective cohort study:	3 yrs.	**a) per 1% HbA_1c_ increase; b) HbA_1c_ ≥6.43% vs. <6.43%; multivariate adjustment.**
	78 Japanese HD patients		All-cause mortality (n = 27): a) HR = 1.11 (0.71–1.74); b) HR = 0.93 (0.34–2.58)
			CV mortality (n = 15): a) HR = 1.04 (0.48–2.28); b) HR = 1.06 (0.16–7.12)
			CVD (n = 23): CVD: a) HR = 1.34 (0.78–2.29); b) HR = 0.81 (0.27–2.46)
**Shima et al.** 2010 [15]	Observational cohort study: 245 Japanese HD patients	43.2 mos.	All-cause mortality (n = 68): HR = 0.712 (0.315–1.609) for HbA_1c_ ≥7% vs. <6.0%; multivariate adjustment.
**Shurraw et al.** 2010 [13]	Retrospective cohort study: 540 Canadian incident HD patients (448 of them with diabetes mellitus)	1.5 yrs.	All-cause mortality (n = 236): a) per 1% HbA_1c_ increase, unadjusted: HR = 1.01 (0.92–1.11); b) HbA_1c_ ≥9% vs. <7%, multivariate adjustment: HR = 1.06 (0.55–2.07).
**Williams et al.** 2006 [14]	Observational cohort study: 24,875 US HD patients	1 yr.	All-cause mortality (15–20%): no clear patterns between HbA_1c_ and death risk; multivariable adjustment.

CV, cardiovascular; CVD cardiovascular disease; HD hemodialysis.

A second heterogeneity between and within the studies derives from the fact that most studies used only one HbA_1c_ value and patients were from mixed cohorts with patients either already under dialysis treatment for various times or at the start of dialysis treatment. To avoid this heterogeneity, we followed a single center cohort of incident dialysis patients from a described geographical region with full-ascertainment of all patients starting dialysis treatment. Furthermore, we did not only use a baseline HbA_1c_ value but all HbA_1c_ values of the entire observation period which resulted in 880 values in total or one value on average every 3.16 months in each patient. This dense network of measurements used in a time-dependent Cox regression modeling has the major advantage that not a single measurement is the basis of the analysis but a complete coverage of the glycemic control during the observation period. In our case this was even extended by not only considering the HbA_1c_ values but also each single measured value of blood pressure, albumin, CRP and hemoglobin during the entire observation period as well as the occurrence of a CVD event before dialysis treatment was initiated.

There are only two other studies which applied time-dependent modeling of HbA_1c_ values in patients with DM. A study of almost 24,000 US hemodialysis patients with DM done by Kalantar-Zadeh et al. [16] followed HbA_1c_ values for 3 years and averaged all measures for each patient during any given calendar quarter. HbA_1c_ values were divided into categories reaching from <5% to ≥10%, and 1% increments in between. They observed lower unadjusted mortality to be associated with poor glycemic control, however, after adjustment for potential confounders the direction of the association changed and higher HbA_1c_ levels were now incrementally associated with higher death risks. They also stated that one-third of all prevalent diabetic hemodialysis patients in the US have HbA_1c_ values within the normal range comparable to the general population. Previous hyperglycemia that caused their micro- or macrovascular disease appeared to be “burnt-out” by complex pathophysiologic mechanisms [16]. Shurraw et al. [13] investigated the association of HbA_1c_ and all-cause mortality in a retrospective cohort of 448 hemodialysis patients with DM. They did not find any association between HbA_1c_ levels and mortality.

Only one interventional study in dialysis patients with DM by McMurray et al. [21] revealed that intensive diabetes education and care management leading to improvements in patient outcomes, glycemic control and a better quality of life. Nonetheless, after a 12-month period no statistically significant difference in survival benefit between the intensive intervention group and control group could be observed. In a prospective interventional study in patients with DM but without renal failure [4] an increased risk of mortality for low HbA_1c_ values could be found, however, up to now there was no study in dialysis patients with DM supporting this finding. Our patients were under long-term observation up to seven years and we might speculate that intensive diabetic control increases the risk for hypoglycemic episodes which with increasing frequency increases the risk to die in the long-run. On the other hand it is interesting that non-survivors who had HbA_1c_ levels below 7% at baseline and during the first 3 months of follow-up died mostly from chronic heart failure and therapy withdrawal. This favors the idea that those non-survivors with low HbA_1c_ died mainly due to a bad general health condition than due to an intensive diabetes control. As an example, weight loss due to an intercurrent illness or malnutrition may by itself lead to lower HbA_1c_ levels. This is in line with our observation that HbA_1c_ below 7% was associated with significantly lower albumin and phosphorus concentrations and higher CRP levels (Table 3). In addition, it is well known that low HbA_1c_ levels in dialysis patients may not be caused by better glycemic control, but a shortened life span of erythrocytes [22], [23]. In both studies, HbA_1c_ correlated positively with hemoglobin levels and negatively with the administered erythropoietin dose. Thus, low HbA_1c_ may also be a consequence of erythropoietin resistance, which in many instances is caused by some intercurrent illness and inflammation.

### Strength and limitations of the study

The prospective recruitment of all patients starting dialysis treatment over a period of six years in a clearly described geographic area allowed a complete ascertainment of incident dialysis patients over a defined period of time with almost no loss to follow-up during a long observation period. Therefore the most important bias of cross-sectional studies with a mix of prevalent and incident cases and the resulting survival bias can be excluded when it comes to survival bias after start of renal replacement therapy. On the other hand it might lack generalizability to other ethnic populations as well as other recruitment procedures. A further limitation of this study is the small sample size which limits the number of variables for which the analysis can be adjusted. However, there were no differences for the age- and sex-adjusted models and the models with extended adjustment. Even if the sample size might have limited the generalizability of our findings, our study might be a stimulus for other studies which have the data with this depth (duration of observation and granularity of data points) available for analysis.

Despite these limitations our study has notable strengths. It is a single center study with uniform laboratory measurements of high frequency and continuity collected over a period of up to seven years. By analyzing HbA_1c_ on risk of mortality, CVD events and the combination of CVD and PAD events in a time-dependent modeling framework, we were able to include all measurements over the whole observation period. Our study is up to now the only inception cohort study with time-dependent measurements over a long observation period.

### Conclusions

Our prospective observational cohort study of patients with DM initiating dialysis treatment considered the entire information collected on HbA_1c_ over a period of more than seven years and observed a significant association between low HbA_1c_ levels and all-cause mortality. Based on the causes of death we suspect that the low HbA_1c_ levels associated with increased mortality were not a consequence of intensive glucose-lowering therapy, but rather caused by poor general health condition. For CVD events and the combination of CVD and PAD events no significant association with HbA_1c_ levels could be observed.
